# Role of Plant-Derived Flavonoids and Their Mechanism in Attenuation of Alzheimer’s and Parkinson’s Diseases: An Update of Recent Data

**DOI:** 10.3390/molecules23040814

**Published:** 2018-04-02

**Authors:** Ghulam Hussain, Longbin Zhang, Azhar Rasul, Haseeb Anwar, Muhammad Umar Sohail, Aroona Razzaq, Nimra Aziz, Asghar Shabbir, Muhammad Ali, Tao Sun

**Affiliations:** 1 Department of Physiology, Faculty of Life Sciences, Government College University, Faisalabad 38000, Pakistan; drhaseebanwar@gcuf.edu.pk (H.A.); umar.sohail@gcuf.edu.pk (M.U.S.); aroonarazzaq@yahoo.com (A.R.); nimra.aziz1794@gmail.com (N.A.); 2 Center for Precision Medicine, School of Medicine and School of Biomedical Sciences, Huaqiao University, Xiamen 361021, China; leocheung@hqu.edu.cn; 3 Department of Zoology, Faculty of Life Sciences, Government College University, Faisalabad 38000, Pakistan; drazharrasul@gmail.com (A.R.); alisam007@hotmail.com; (M.A.); 4 Department of Biosciences, COMSATS Institute of Information Technology, Islamabad 44000, Pakistan; asghar.shabbir@comsats.edu.pk

**Keywords:** flavonoids, natural compounds, biomolecules, neurodegenerative diseases, nitric oxide, tumor necrosis factor-α, tyrosine kinase, monoamine oxidase

## Abstract

Neurodegeneration is a progressive loss of neuronal cells in certain regions of the brain. Most of the neurodegenerative disorders (NDDs) share the communal characteristic such as damage or reduction of various cell types typically including astrocytes and microglial activity. Several compounds are being trialed to treat NDDs but they possess solitary symptomatic advantages along with copious side effects. The finding of more enthralling and captivating compounds to suspend and standstill the pathology of NDDs will be considered as a hallmark of present times. Phytochemicals possess the potential to alternate the synthetic line of therapy against NDDs. The present review explores the potential efficacy of plant-derived flavonoids against most common NDDs including Alzheimer’s disease (AD) and Parkinson’s disease (PD). Flavonoids are biologically active phytochemicals which possess potential pharmacological effects, including antiviral, anti-allergic, antiplatelet, anti-inflammatory, anti-tumor, anti-apoptotic and anti-oxidant effects and are able to attenuate the pathology of various NDDs through down-regulating the nitric oxide (NO) production, by reducing the tumor necrosis factor-α (TNF-α), by reducing the excitotoxicity of superoxide as well as acting as tyrosine kinase (TK) and monoamine oxidase (MAO) inhibiting enzyme.

## 1. Introduction

Neurodegeneration is a composite progression of progressive loss of both the function and structure of neurons and involves the muscle weakening and deterioration of innumerable physiological functions of the body [1,2]. Deregulated lipid metabolism is one of the hallmarks of degeneration in the majority of neurodegenerative disorders [3,4,5,6]. During neurodegeneration, cell death is preeminent in post-mitotic cells, with an enormous number of neurons eliciting apoptotic signals, which might be the consequence of oxidative stress [7]. Both intrinsic and extrinsic pathways are two major aspects of apoptosis which are associated with mitochondrial and plasma membrane receptors, respectively [8]. Proteins of bcl-2 family play a crucial role in regulating the pathways of apoptosis involving mitochondria. They can be categorized into two functionally diverse groups as pro- and anti-apoptotic proteins [9]. Neurodegenerative diseases (NDDs) usually involve the discerning loss of neurons as well as engrossment of diversified functional systems describing their clinical presentation expanded by genetic, biochemical and molecular pathological factors. Enormous studies have revealed the deposition of proteins with transformed physiochemical properties within the human brains in NDDs [10]. Aggregation of interrelated proteins serves as a major hallmark of the NDDs, suggesting the same pathophysiology of the degenerative process. Recent studies state that such proteinopathies expose the contribution of the same protein in a number of diseases, thus signifying a common pathological progression [11].

Neurodegenerative diseases such as Alzheimer’s disease (AD), Parkinson’s disease (PD), Huntington disease (HD), Schizophrenia, Amyotrophic Lateral Sclerosis (ALS), seizure disorders, and head injuries are foremost health issues along with other systemic disorders [12,13,14]. Various studies also state the involvement of oxidative stress in the pathophysiology of NDDs. Oxidative stress causes the neuronal cell death by inducing the neuronal damage and modulating the intracellular signaling [15].

Natural products persist as a promising source of immense chemical diversity, biochemical specificity and various molecular characteristics which make them suitable for the modulation of multiple signaling pathways/cascades in various pathological conditions such as cancer and neurodegnerative diseases [16,17,18,19,20,21,22]. Currently, phytochemicals including flavonoids, alkaloids, terpenoids, and phenols are of considerable interest for the treatment of such diseases [12]. We have recently reviewed protective roles of plant-derived alkaloids in neurodegenerative diseases [23]. Flavonoids have been sanctioned to activate neuronal endogenous anti-oxidant status, thus, shielding them from neurodegeneration. Neuroprotective mechanism of flavonoids proceeds via suppression of lipid peroxidation, inhibition of inflammatory mediators, modulation of gene expressions and activation of anti-oxidant enzymes which makes them ideal therapeutic representative for the treatment of NDDs [24].

This review intends to emphasize the molecular mechanism of plant-derived flavonoids to diminish the risk of cellular degeneration and to enhance cell survivability. The scientific basis underlying the neuroprotective effect of this novel class of phytochemicals has been brought to light. This will facilitate the understanding of researchers regarding the pharmacological role of flavonoids in NDDs, thus, suggesting areas for further research. The literature was screened through various e-sites, including Springer Link, PubMed, Elsevier Science Direct Scopus and other relevant medical journals, highlighting the updates in this area of research. Key words used for searching are “Flavonoids”, “Neurodegenerative Diseases”, “Alzheimer’s disease (AD)”, and “Parkinson’s disease (PD)”.

## 2. Alzheimer’s Disease

Alzheimer’s disease (AD) is the progressive weakening of cognitive functions, memory, and learning [25], characterized by the aggregation of β-amyloid (Aβ) peptide, tau protein hyperphosphorylation, and amplified oxidative stress. However, the reasons for the massive majority of sporadic forms of AD remain un-demarcated [26]. Aβ peptides primarily form the senile plaques in the affected brain areas and these areas in turn exhibit a reduced number of synapses. These plaques usually contain scratched neurons, signifying the neuritis and synapse damage by Aβ. Aβ_40/42_ is generated by gamma-secretase-mediated sequential cleavages of the amyloid precursor protein (APP) and β-secreatase-(beta-site amyloid precursor protein cleaving enzyme, BACE) [27]. Hyperphosphorylated tau and Aβ accumulation in the brain are proposed to play an important role in the neurodegenerative process of AD [28] by activating the neuronal damage. Moreover, oxidative stress is another hallmark of AD along with the Aβ accumulation and hyperphosphorylation of tau [29]. In the pathogenesis of AD, oxidative stress may be the earliest change to occur [30]. Oxidative stress may be caused by hypercholesterolemia through forming reactive oxygen species (ROS) [31]. Endoplasmic reticulum (ER) stress can also be triggered by oxidative stress, and sustained ER stress can lead to the additional oxidative damage [32]. Currently, the global prevalence of dementia is as high as 36 million and is expected to reach 66 million by 2030 and 115 million by 2050, with almost two-third of the patients from the developed countries [33].

## 3. Parkinson’s Disease

Parkinson’s disease (PD) is characterized by the loss of dopaminergic neurons in the substantia nigra (SN) [34]. Initial symptoms of the disease include slowness of movement, shaking, rigidity, difficulty with walking, and behavioral problems [35]. It is a late onset disorder that occurs in 1–2% people over the age of 60 years [36]. The distinctive neuropathological changes in the brain include the abnormal formation of Lewy bodies. Degenerated dopaminergic nigrostriatal neurons with the Lewy bodies are major neuropathological correlation of motor damage in PD, but noradrenergic, adrenergic, glutamatergic, cholinergic, and GABAergic nerve cells also show identical damage in cytoskeleton [37]. Dopaminergic neurons of SN are progressively and selectively degenerated [38]. Neuroinflammation and particularly, microglial activation is associated with the pathogenesis of PD [35]. Microglial activation triggers the formation of a broad range of cytotoxic factors, including interleukin-1β (IL-1β), nitric oxide (NO), ROS, and tumor necrosis factor-α (TNF-α), causing neurodegeneration [39]. The adult hippocampal dentate gyrus (DG) receives inputs from dopaminergic neurons in the SN. So, deterioration of dopaminergic neurons may directly affect adult hippocampal neurogenesis [40].

## 4. Phytochemicals

Phytochemicals are a diversified group of bioactive compounds naturally occurring in plants. Various classes of phytochemicals including flavonoids, alkaloids, terpenoids, and phenols act as protective agents in nervous system disorders [41]. Phytochemical therapies have been extensively used against neural symptoms, but the underlying mechanism of action of phytomedicines is yet to be determined. One of the mechanistic approaches of phytomedicines is their potential efficacy to act as anti-oxidant and anti-inflammatory agents [42]. A copious number of phytochemicals are able to alter the neuronal excitability via inhibiting or activating the ion channels or specific receptors [41]. In this article, we have reviewed the potential efficacy of flavonoids as neuroprotective agents against NDDs by specifically focusing on molecular interactions of these compounds with various cellular targets.

## 5. Flavonoids

Flavonoids are naturally occurring, biologically active, and therapeutically effective polyphenols abundantly found in fruits and vegetables. They are classified in several categories including flavanols, flavonols, flavones, flavanones, isoflavones, anthocyanidins, and chalcones based on their chemical structure. To date, over 9000 flavonoids have been well-known, mainly found in fruits, vegetables, and beverages (tea, coffee, beer, wine and fruit drinks). Flavonoids and their metabolites exert countless health promoting effects both in human and animals. They possess multiple biological effects such as antiviral, anti-allergic, antiplatelet, anti-inflammatory, antitumor, and antioxidant activities [43]. Moreover, they can cross the blood-brain-barrier (BBB) and may exhibit neuropharmacological activities at the molecular level, influencing the protein function and gene expression. Importantly, dietary intake of flavonoids up-regulates the brain derived neurotrophic factor (BDNF) and thus improves the performance of spatial memory [44]. Extensive evidences have suggested their role in the attenuation of pathological pathways of NDDs [45,46]. Diet and lifestyle may play a potential role in the improvement of cognitive function and can also delay the onset of age related health disorders. Importantly, flavonoids enriched foods can induce memory and cognition improvements both in animals and humans. Similarly, it is proposed, by a growing number of studies, that dietary intervention with particularly diet rich in polyphenols exert neuroprotective effects in the brain, including the protection of neurons against neurotoxin-induced injury, suppression of neuroinflammation, and also has a potential to promote cognitive, learning, and memory functions [47]. Furthermore, flavonoids can modulate the immune system of brain, attenuate the neuroinflammation by inhibiting the production of nitric oxide and cytokines induced by activated microglia [48]. Thus, flavonoids signify their importance as potent molecules in the pursuit to develop a new group of drugs, having the ability to counteract the neuroinflammation and NDDs.

Hence, multiple effects of flavonoids have drawn the interests of scientists towards the investigation of neuroprotective role of flavonoids. Classifications of flavonoids with their dietary sources are represented in Table 1.

## 6. Classes of Flavonoids and Their Implications in Neurodegenerative Diseases

**Figure 1:**
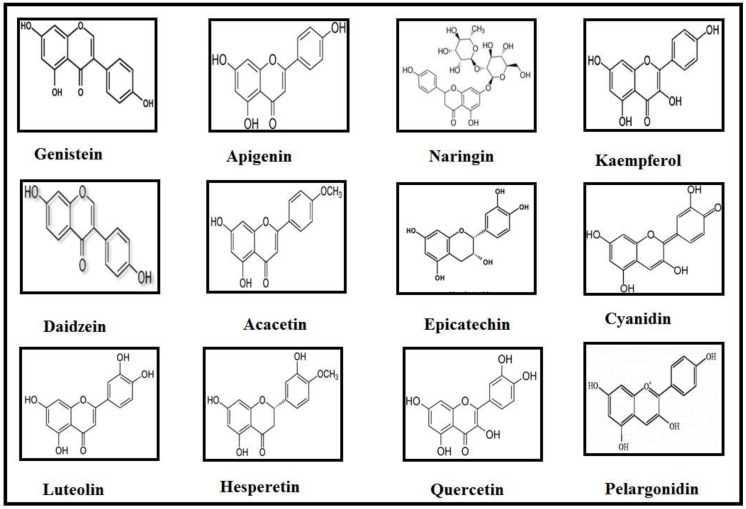
Structures of compounds that discussed in this review.

**Figure 2:**
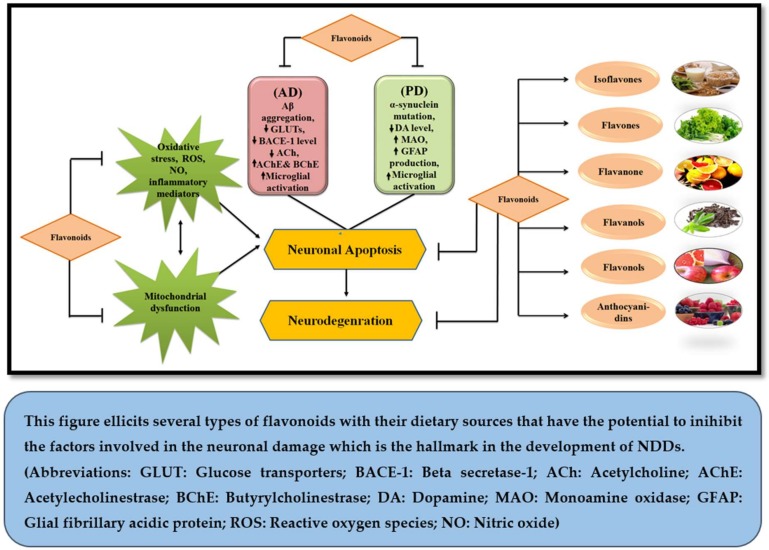
Neuroprotective potential of flavonoids.

Classification of Flavonoids is based on their tertiary structure (Figure 1) and growing number of evidences have strengthened the idea that they may bestow attenuating effects against neurological, neurodegenerative, psychological and other diseases (Figure 2). In the present effort, we have reviewed the mechanisms and effects of flavonoids on Alzheimer’s disease and Parkinson’s disease based on the availability of published data.

## 7. Isoflavones

### 7.1. Genistein

Genistein (Gen) is a primary soybean isoflavone, which exhibits numerous beneficial aptitudes for human health. It has structural similarity with endogenous steroid estrogen which enables it to mimic the pharmacological action of estrogen [67]. Gen may potentially reduce the process of neurodegeneration followed by inflammation by hindering the microglial inflammatory reactions in response to the exogenous stimulus [68]. A cumulative number of studies propose that Gen acts as a protective agent for neurons and thus it efficiently elicits a neuroprotective response in amyotrophic lateral sclerosis [69]. Importantly, it also shelters the cortical neurons of the human brain against free radical damage and thus portrays its anti-oxidative as well as anti-inflammatory property [70]. Moreover, its neuro-protective efficacy in NDDs has been discussed as follows.

#### 7.1.1. Genistein in PD

PD is followed by the continuous damage of dopaminergic neurons in substantia nigra which ventures to the striatum [71]. Evidence from the imaging studies also report the minimized levels of dopamine in fronto-striatal circuit in PD patients [72]. Hence, it can be declared that the loss of dopaminergic neurons crucially upholds the underlying pathogenesis of PD. Gen has potential to protect the dopaminergic neurons in a dose-dependent manner against lipopolysaccharide (LPS)-induced neurotoxicity. It inhibits the production of NO, TNF-α, and superoxide in microglia as well as in mesencephalic neuron-glia cultures [70]. Moreover, the chief immune cells of brain microglia are eagerly activated in response to any infection or injury which leads to the release of pro-inflammatory factors [68] like NO and superoxide [73], which may form complexes with proteins causing the alteration of their functions and eventually causing cell death. Gen can attenuate the production and accumulation of superoxide and NO, thus delivering its neuro-protective efficiency to dopaminergic neurons and sheltering the dopaminergic neurons from a post injury response [70,73]. The least effective dose of Gen has been found as 0.25 µM while at 50 µM concentration, it is proposed to elucidate the toxicity in neuronal glia cultures. Interestingly, it fails to block the pro-inflammatory factors in glial cell cultures at the dose of 2.5 µM followed by the LPS induction in an animal model study [68]. Furthermore, it is obligatory to explore the other molecular targets involved in neuroprotection offered by Gen in the future.

#### 7.1.2. Genistein in AD

Aggregation of Aβ proteins has been crucially involved in the pathogenesis of AD and it also acts as the foremost target for therapeutic development of the disease [74]. Aβ-induced neuronal cell death is also a leading cause of AD pathogenesis [75]. Gen is the foremost phytoestrogen in soybean and proficiently mimics the pharmacological functions of estrogen [67]. Estrogen possesses an affirmative potential of blocking the Aβ-induced neuronal cell death [75]. Gen possesses impartial neuroprotective potential because of its capability to act as estrogen receptors (ERs) agonist as they mediate the defensive cascade against Aβ-induced toxicity [76]. It also attenuates the crucial clinical outcome of memory impairment in AD patients by protecting the neuronal network of the brain. Hence, estrogen is also involved in memory and learning development in numerous brain regions (hippocampus, neo-cortex, nuclei of the basal forebrain) [77]. Moreover, Gen at 0.375 μg/mL dose protected the rat hippocampal neuronal cells by up-regulating the protein kinase signaling pathways [78]. It also has a capability to decrease the production of ROS at 50 μM and thus portrays its role as an anti-oxidant agent. The underlying mechanism to this protective feature of Gen involves the inhibition of mitochondrial transition pore opening, which ultimately prohibits the mitochondrial release of ROS in β-amyloid peptides 25–35-induced PC12 cells [79]. Importantly, it does not provide neuroprotection at the dose of 0.1 or 100 nM [77]. Several studies reveal that it does not elicit proliferative side effects on uterine endometrial cells along with the blocking of acetylcholine-induced neurotoxicity. Therefore, Gen may be a beneficial mediator for the treatment of AD [77]. Lastly, although Gen has been reported to possess neuroprotective activities, but still, there is a lack of clinical studies on its application as a therapeutic agent [80].

### 7.2. Daidzein

Daidzein belongs to the isoflavones class of flavonoids, naturally occurring entirely in legumes and soybeans [81]. Daidzein along with other isoflavones have been found in many plants like Kudzu (*Pueraria lobata*) and Kwao Krua (*Pueraria mirifica*) produced by the secondary metabolism of phenylpropanoid pathway [82]. Daidzein possesses diversified biological effects in various biological systems and may be able to serve as an agent to prove the therapeutic efficacy of flavonoids against several health issues [83] including improvement in blood cholesterol level, osteoporosis reduction [84], attenuating the risk of certain hormone related cancer, and coronary heart diseases [85]. Moreover, it also efficiently employs its action as neuroprotective agent via acting as agonist of estrogen [81]. It has the capability to bind with ERs in brain because of its structural similarity with estrogen. Thus, it elucidates ER-dependent activation of estrogen receptive promoters and DNA binding in numerous cell types [86]. ERα and ERβ are two prime types of estrogen receptors which are expressed in brain and daidzein parades more affinity of binding with ERβ as compared to ERα [87]. Therefore, it can portray a potential role in the attenuation of various NDDs as explained bellow.

#### Daidzein in PD

As the pro-inflammatory factors and microglia activation have been supposed to play a crucial role in neuronal cell death concomitant with PD [73]. Thus inhibition of both factors has been known to be associated with the possession of neuroprotective properties in PD [88]. Daidzein exhibits an effective property to diminish the release of inflammatory mediators in BV-2 microglial cells induced by lipopolysaccharide (LPS) [89]. An experimental investigation on male Sprague–Dawley rats reveals that daidzein exerts pro-oxidant activity as well as significantly attenuates malondialdehyde (MDA) content in the brain at an oral dose of 2 and 20 mg/day for 4 weeks dissolved in corn oil [90]. Whereas, another study suggests that daidzein may persuade detrimental effects at high concentration [91] and elucidates oxidant properties rather than anti-oxidant action by affecting the antioxidant enzyme defense system in rat hepatoma H4IIE cells [92]. Overload of free radicals followed by the oxidative stress is one of the most common features of neurodegenerative diseases such as PD and AD. It results in the production of ROS and NO which affect biosystem of body and has been found to affect the function and structure of neural cells. Thus it contributes to an extensive range of NDDs including AD and PD [93]. Daidzein inhibits oxidative stress associated production of NO and ROS [94]. Therefore, anti-oxidant agent which efficiently removes the ROS could portray potential therapeutic effect against PD [93]. Furthermore, studies on LPS-stimulated microglial cells suggest that it elucidates neuro-protective potential because of its efficiency to inhibit the microglia activation and ensuring the release of soluble pro-inflammatory factors [88]. Despite exhibiting the multiple aspects in neuroprotection there is still a dearth of clinically proven consideration and medication of daidzein.

## 8. Flavones

### 8.1. Luteolin

Luteolin (3′,4′,5,7-tetrahydroxyflavone) belongs to the flavone group of flavonoids which is abundantly found in the plant kingdom [95]. Chemical structure of luteolin comprises of C_6_-C_3_-C_6_ structure and contains an oxygen-containing ring, two benzene rings, and 2–3 carbon double bond [96]. It is abundantly found in fruits and vegetables such as chrysanthemum flowers, apple skins, cabbage, peppers, carrot, leaves of onion, broccoli, parsley, and celery [50,51,52]. It is well known for its potential anti-inflammatory and anti-oxidative properties and also exhibits phytoestrogen like activities [97,98]. Plants enriched with luteolin are utilized against cancer, inflammatory diseases and hypertension in Chinese traditional medicine [95].

#### 8.1.1. Luteolin in AD

AD is the most common neurodegenerative disease which leads to the development of senile dementia. Cognitive dysfunction is particularly caused by AD. It is reported that cognitive dysfunction in cerebral hypoperfused rats can be protected by luteolin at a dose of 150 and 450 mg/kg [99]. The hallmark in the pathology of AD is Aβ plaques formation. To find out the Aβ reducing capability of luteolin, primary neuronal cells which are the SweAPP-overexpressing mice were treated with luteolin and it was seen that it momentously lessened the Aβ generation [100]. The mechanism behind the reduction of Aβ generation may encompass the GSK-3α isoform selective inactivation that enhances the p-PS1 levels which is the γ-secretase complex catalytic core [101]. Recently, it has been found that AD pathologies in mice, persuaded by traumatic brain injury can be reduced by luteolin at the dose of 20 mg/kg/day for 15 days [102]. Luteolin also possesses the protective effects on structure of hippocampus and learning flaws in streptozotocin-stimulated Alzheimer’s rat model. The administration of luteolin at10 and 20 mg/kg dose [101], shows significant results in this context.

#### 8.1.2. Luteolin in PD

It is a well-known fact that the level of dopamine (DA) is reduced in SN in case of PD. In addition to the reduced level of DA, the inflammation in the brain accompanied by over-activation of microglia is also involved in the pathology of PD [103,104,105]. Unnecessary quantities of cytotoxic and pro-inflammatory factors produced by the activation of microglia in substantia nigra are lethal to neurons [106]. It was reported by an in vitro investigation on luteolin that its (5 µM) treatment may protect the LPS-induced dopaminergic neuronal degeneration by inhibiting the activation of microglia [107]. However, very limited work has been done to explore the beneficial effect of luteolin on CNS.

### 8.2. Apigenin

Apigenin (4′,5,7,-trihydroxyflavone), a naturally occurring phytochemical, belongs to flavone group of flavonoids. Naturally, it can be extracted from flowers and buds of *Hypericum perforatum*. It is copiously found in common vegetables and fruits such as onion, parsley, grapefruit, and orange [53,108]. It exhibits multiple pharmacological effects such as anti-inflammatory, anti-apoptotic, anti-oxidative, purgative, antiviral, and anti-mutagenic [109,110]. It has been shown that apigenin can reduce glutamate-induced Ca^2+^ signaling in murine cortical neurons [111].

#### 8.2.1. Apigenin in AD

One of the pathogenic symbols of AD is Aβ generation, aggravated due to mutations in APP [112]. Furthermore, the buildup of Aβ leads to the microglial over-activation around Aβ plaques [113]. Neurotoxicity of Aβ can be induced by free radical production caused by transition metals like copper. Treatment with 10 µM apigenin can cease the enhanced expression of Aβ precursor protein caused by copper but it is not effective at any other concentration [114]. Apigenin also possesses the ability to improve the memory impairment associated with AD, to prevent oxidative stress and to decrease the burden of Aβ plaques. Numerous studies have demonstrated the anti-inflammatory [115] and anti-apoptotic effects of apigenin in various animal models [110] as well as in human [116]. It is reported that apigenin protects neurons against inflammatory stress and limits apoptotic cell death as well as reduces the neuronal hyper-excitability [53]. Furthermore, it was demonstrated that apigenin could inhibit the activation of pro-inflammatory cytokines and NO production, protecting AD neurons from inflammatory-induced stress. The concentration of apigenin 50 µM (IC_50_ value) can protect neurons against neurite shortening and neuronal death as well as reduce apoptosis [111]. Moreover, the IC_50_ values of apigenin ranging between 10 and 100 µM can be able to reduce the production of NO and pro-inflammatory cytokines [115]. It has been shown that 10 mg/kg and 20 mg/kg intraperitoneal administration of apigenin reduces the activity of AChE [117], which is a key enzyme involved in the development of AD. All these investigations suggest that apigenin has the ability to overcome the progression of AD. Hence, it needs to be introduced in clinical trials as well.

#### 8.2.2. Apigenin in PD

The primary indications of PD are shakiness, postural abnormalities, bradykinesia, muscular rigidity, and tremor at rest [118]. A well-known hallmark in the pathology of PD is neuronal inflammation-induced glial cell activity in the SN [119]. The available treatment is DA agonist but the chronic administration of _L_DOPA or DA agonist can lead to severe non-motor and motor adverse effects [120]. The worse effect of _L_DOPA or DA agonist diverts the interest of scientists towards phytomedicines for treatment of PD to reduce or prevent the adverse effects. It was shown that apigenin enhanced the locomotor capability and proved to be very effective in a dose-dependent manner (5, 10 and 20 mg/kg) [121]. In vitro investigation has suggested that apigenin exerts inhibitory property against inflammatory mediators, proposes that it may possess neuroprotective potential against inflammation mediated diseases such as NDDs [122]. It was also reported that apigenin could secure the dopaminergic neuronal loss in Parkinson’s mice model by attenuating the microglial activation and neuroinflammation at dose of 10 and 20 mg/kg [121]. Importantly, administration of apigenin expressively prohibits the neuroinflammation in SN [117]. At present, the treatment of PD is dependent on DA agonists. There is a dire need to introduce apigenin in preclinical trials for the treatment of PD to overcome adverse effects of currently used medicines.

### 8.3. Acacetin

Besides luteolin and apigenin, there is another flavonoid compound known as acacetin (5,7-dihydroxy-4-methoxyflavone), which belongs to flavone group of flavonoids. It is extracted from *Clerodendrum inerme* (L.) Gaertn (CI) which possesses potential therapeutic efficacy against neuropsychiatric disorders [123]. It also exerts several biological actions including anticarcinogenic, anti-inflammatory, and antioxidant actions [124,125,126]. It can also be extracted from *R. pseudoacacia* [55]. The antioxidant and anti-inflammatory role of acacetin give the direction that it can be beneficial in the treatment of NDDs such as AD and PD.

#### 8.3.1. Acacetin in AD

Neuroinflammation is one of the hallmark in the pathology of AD. Activation of microglia plays a crucial role in neurodegeneration mediated by inflammation. Microglial over-activation can lead to the neuronal cell death and CNS disorders through the production of several cytotoxic and pro-inflammatory factors such as IL-1β and TNF-α [127,128]. The transcription factor known as nuclear factor-κB (NF-κB) regulates the expression IL-1β, TNF-α, and iNOS [129]. Mitogen activated protein kinases (MAPKs) including JNK and p38 are also found to be involved in the microglial-induced inflammation [130,131]. It has been demonstrated that acacetin can inhibit the NO release and attenuates the IL-1β and TNF-α. Importantly, acacetin inhibits the p38 MAPK and NF-κB activation. Experimentation on the mouse model of lipopolysaccharide (LPS) mediated neuroinflammation indicated that acacetin expressively suppressed the activation of microglia in a dose-dependent manner [55]. Another factor which is involved in the neurodegeneration is excitotoxicity caused by excessive glutamatergic neurotransmission via NMDAR [132]. Glutamate is the chief excitatory neurotransmitter in the CNS and plays a crucial role in memory, learning, and cognition. In addition, excessive release of glutamate enhances the levels of intracellular Ca^+^ which in turn enhances the production of free radicals and mitochondrial dysfunction and eventually causes neuronal damage. It shows that acacetin inhibits the release of glutamate. So, the inhibition of glutamate ultimately stops the cascade of damaging cellular processes [133]. The inhibiting properties of acacetin indicate that it can be beneficial in the treatment of NDDs, particularly AD.

#### 8.3.2. Acacetin in PD

Neuroinflammation is considered to be the most prevalent factor involved in the pathology of PD. Studies show that acacetin inhibits the inflammatory factors production and hence protects the dopaminergic neurons, major targets in the development of PD [54]. More work is needed to be done to ensure the therapeutic role of acacetin in context of PD.

## 9. Flavanones

### 9.1. Hesperetin

Hesperetin (3′,5,7-trihydroxy-4-methoxyflavanone) belongs to the flavanone class of flavonoids, found in citrus fruits [56]. It is derived from the hydrolysis of aglycone, hesperidin (hesperetin 7-rhammnoglucoside) [134]. It exerts neuroprotective effects by acting as anti-inflammatory and anti-oxidative agent [135].

#### Hesperetin in AD

Aβ deposition results in prevention of insulin signaling in neurons and reduction in membrane insulin receptor (IR) activity which leads to the reduction in insulin levels and glucose transporters (GLUTs) in brains of AD patients [136]. Deposition of Aβ_25–35_ impairs glucose uptake and also leads to the neuronal damage by cellular autophagy. Hesperetin at a dose of 97.2 µM protects against Aβ_25–35_-stimulated neuronal damage [137]. It also possesses the ability to ameliorate the Aβ impaired glucose uptake moderately by impeding autophagy. The suggested dose of hesperetin which is very effective in attenuating the neuronal autophagy is 1–20 µM [138]. Importantly, like Aβ aggregation, oxidative damage which is induced by the lipid peroxidation is another feature involved in the pathophysiology of AD. It was demonstrated that with the IC_50_ values of 179.1 µM, hesperetin intensely inhibited the lipid peroxidation that could cause oxidative damage [137,138].

### 9.2. Naringin

Naringin is a flavanone glycoside derived from naringenin (a flavonoid) and it is one of the chief active constituents of Chinese herbal medicines including *Citrus medica* L. (CM), *Citrus aurantium* L. (CA), and *Drynaria fortunei* (Kunze) J. Sm. (DF) [139,140]. It is found in citrus fruits such as grapefruits [141] and bitter taste of citrus juices is dedicated to this flavonoid [142]. It executes several pharmacological and biological effects including anti-carcinogenic, anti-osteoporotic, anti-ulcer, anti-apoptotic [143], anti-inflammatory, cholesterol reducing and antioxidant effects [144].

#### Naringin in PD

PD is partially caused by microglial activation which are native immune cells of brain. Microglial activation is resultant of DA neuronal damage [145]. The activated microglia may also produce several neurotoxins such as pro-inflammatory cytokines and inducible nitric oxide synthase (iNOS) which leads to the nigrostriatal DA neuronal cell death [146,147]. Recently, it was reported that oral administration of naringin at the dose of 100 mg/kg reduced the microglial activation by decreasing the expression of glial fibrillary acidic protein (GFAP) [148]. GFAP expression is reported to be altered following brain damage as in case of PD [149]. It was mentioned that oxidative stress and neuroinflammation are involved in the pathology of PD. It was proposed that oral administration of 80 mg/kg naringin (dissolved in 0.5 mL of 0.25% Sodium carboxymethylcellulose) for two weeks in 3-nitropropionic acid-induced neurodegeneration rat models modulated the inflammatory reactions and oxidative stress, thereby, giving an idea of its neuroprotective effects against neurodegeneration [150]. Additionally, naringin also exerts neuroprotective effects by the initiation of neurotrophic factors [145,151,152]. Furthermore, it has been reported that naringin enhances the GDNF level in neurotoxin model of DA neurons and it also reduces the level of tumor necrosis factor-α in microglia. The effective dose reported in this aspect is 80 mg/kg (suspended in 0.25% sodium carboxymethylcellulose that was dissolved in 0.9% saline) [151]. Thus, these indications propose that naringin might be a possible natural compound involved in the treatment and anticipation of the NDDs.

## 10. Flavanols

### 10.1. (−) Epigallocatechingallate

(−) Epigallocatechin gallate (EGCG) contains 3 phenol ring structure and is one of the type of catechin. It is the main bioactive component of green tea leaves while it is also found in black tea in a minor quantity [43]. Polyphenols from green tea including ECCG have been reported to exert anti-oxidant [153], anti-carcinogenic [154] and anti-inflammatory effects [155]. EGCG is a major constituent of green tea that is responsible for its health-promoting potentials. The presence of two tri-phenolic groups in its structure is associated with its stronger activity [156]. Furthermore, its anti-oxidant activity has capability to attenuate neurotoxicity as well as neuronal damage resulting from the free radicals attack [157].

#### (−) Epigallocatechingallate in AD

Neurotoxicity of Aβ and neuronal cell death via an apoptotic procedure is a well-known hallmark of AD which is mediated by the production of free radicals and the state of pathogenesis could be accomplished through free radicals scavengers and anti-oxidants [158]. EGCG acts as a potent anti-oxidant agent and prevents the hippocampal neuronal cell death [153]. Programmed cell death, apoptosis, is reported as the distinct process of cell elimination from necrotic cell death. Caspase activation, most importantly, leads neuronal cells towards apoptosis [159]. Thus, caspase might play a crucial proliferative role in Aβ-induced neuronal cell death. Interestingly, EGCG obstructs the augmented caspase activity induced by Aβ_25–35_ and thus can attenuate apoptosis in neuronal cells via rummaging the ROS [160]. Moreover, it also attenuates the major hallmarks of AD pathology such as the interaction between ROS, apoptosis, and Aβ which chiefly contribute to the neuronal cell death. Most importantly, consumption of green tea may reduce the risk of AD [113] and its clinical significance has also been revealed by the animal model studies that EGCG can cross the blood-brain barrier (BBB) and can reach the brain parenchyma [156]. 

### 10.2. (−) Epicatechin

(−) Epicatechin (EC), a plant-derived flavanol, naturally found in blueberries, tea, cocoa, and grapes [59]. It has been recognized as a bioactive flavanol which can cross the BBB and absorbed into circulation after digestion of flavanol-rich foods [161,162]. EC has capability to enhance the cardiovascular function and the cortical blood flow especially in the hippocampus, thus, it may facilitate the neurogenesis [59]. Furthermore, its neuroprotective property in NDDs is discussed as follows.

#### (−) Epicatechin in PD

Neuroinflammation plays a very important role in the PD pathogenesis as supported by various human and animal studies which have enlighten the role of inflammatory cascade and oxidative stress in the progression of PD. Oxidative stress induced by the increased production of NO, ROS and thus can causes the nigral cell death [163]. A postmortem tissue study revealed that the oxidative stress-induced NO, ROS and decreased mitochondrial activity are chiefly involved in pathogenesis of PD [164] suggesting that the agents which hinder the production of NO and ROS and also able to favor the decreased mitochondrial activity might play a protective role in PD [165]. Green tea polyphenols (GTP) including EC moderately protected the dopaminergic neurons by modifying the NO and ROS levels, conserving the free radical as well as prevent an increase in nitrate/nitrite levels in rat model of PD [166]. ROS induce lipid peroxidation, damage to the mitochondrial membrane and thus disrupt the Ca^2+^ homeostasis [167]. Interestingly, GTP impedes the altitude of NO by stabilizing the Ca^2+^ homeostasis [166] and thus, it could serve as a potential marker to attenuate the pathogenesis of PD. 

## 11. Flavonols

### 11.1. Quercetin

Quercetin (3,3′,4′,5,7-pentahydroxylflavone) belongs to the flavonol class of flavonoids [168], ubiquitously found in apples, onions, tea, red wines, and berries [60,61]. It is also present in medicinal plants such as *Sambucus canadensis* (Elder), *Hypericum perforatum* (St. John’s Wort), and *Ginkgo biloba* [169]. It possesses several pharmacological effects such as vasodilation, anti-inflammatory, and anti-oxidative properties [170]. It also exerts anticarcinogenic, antihypertensive, and antithrombic effects [171]. Moreover, it consistently promotes neuroprotective effects [172] and upsurges the hindrance of neurons to oxidative stress and excitotoxicity by tempering the cell death mechanisms [173,174]. It exerts valuable effects on CNS including cognition development and anti-anxiety effects by the inhibition or stimulation of signal transduction pathways or enzyme activities [175].

#### Quercetin in AD

AD is the most prevalent cause of dementia, characterized by the liberal deterioration in cognitive function. It is noteworthy that quercetin promotes the neuroprotective effects by ameliorating the memory impairment and neuronal cell death [176]. Quercetin also attenuates Aβ aggregation and declines the level of BACE-1 which mediates the cleavage of APP [177]. Furthermore, quercetin significantly protects the neuronal cells from neurotoxicity induced by oxidative stress in case of AD [178]. In vitro study reveals that quercetin acts as antioxidant at low doses (5 and 10 µM) while at high doses (20 and 40 µM) it can cause toxicity [179].

### 11.2. Kaempferol

Kaempferol (3,4,5,7,-tetrahydroxyflavone) is a phytoestrogen, and one of the most usual dietary flavonoids. It is frequently found in tea, broccoli, apples, beans, strawberries, and grapefruits [62,63]. It is known to possess potential anti-inflammatory and anti-oxidative effects [180]. It possesses efficient neuroprotective effects against numerous necrosis and apoptosis-inducing damages such as oxidizing low-density lipoproteins [181,182]. It effectively obstructs the upsurge in ROS which is linked to the oxidative stress [183]. 

#### Kaempferol in PD

Lipid peroxidation is the most common pathological symbol in the development of NDDs. It leads to the occurrence of oxidative damage which is caused by the generation of ROS. It was reported that kaempferol protected the brain against damage caused by ROS at the dose of 30 µM in rotenone-induced acute toxicity model [184]. Like lipid peroxidation, monoamine oxidase-A (MAO-A) also promotes the formation of ROS, causing neuronal cell death [185,186]. It is noteworthy that kaempferol possesses MAO-A inhibiting property at the IC_50_ value of 7 × 10^−7^ M which might be beneficial in the treatment of PD [187]. Additionally, it was proposed through experimentation that kaempferol administration amended motor synchronization, enhanced striatal DA in a dose-dependent manner (25, 50 and 100 mg/kg) [188]. Hence, it is proposed to have anti-parkinsonism properties.

## 12. Anthocyanidin

### 12.1. Cyanidin

Cyanidin-3-glucoside (C3G), is a naturally occurring anthocyanin, mainly found in enormous type of red berries including cranberry, blueberry, blackberry, mulberries, acai berry, and raspberry [64]. Out of which mulberries contain high concentration of anthocyanin and have been traditionally used to prevent and treat the diabetes. Importantly, its root bark has been used as an antitussive, anti-inflammatory, anti-pyretic and diuretic [189]. Furthermore, C3G extracted from mulberry fruit possesses neuroprotective property against glutamate-induced as well as oxygen-glucose deprived neuronal cell death [190,191]. Neuroprotective properties of C3G have been discussed as follows.

#### Cyanidin in AD

C3G is able to neutralize the level of Aβ_1–42_ peptides and minimize the H_2_O_2_-induced neurotoxicity [192,193,194]. More recently, it has also been shown that C3G significantly attenuates the Aβ_25–35_-induced expression of ER stress proteins, loss of cell viability and also tends to reduce the intracellular production of ROS in SK-N-SH cells [195]. It can cross the BBB and tempers the age-related deficits in neurons [194]. C3G, during an in vitro investigation, at 50 and 100 µM is reported to reduce the Aβ_25–35_ oligomer toxicity whereas at 100 µM it significantly decreases the necrotic cell (~44%) formation and apoptosis (~38%) induced by Aβ peptides [196]. Its polyphenolic ring structure seems to be fairly appropriate for precise aromatic connections with aromatic deposits of Aβ_1–42_ [197]. Furthermore, the property to block the Aβ_1–42_ interaction with the neuronal plasma membrane was also offset by the C3G. In this repute, several studies propose the adherence of soluble oligomeric Aβ_1–42_ peptides to plasma membrane causing lesions by a combination of impermeable pores formation and lipid peroxidation and thus finally leading to the cell death [198]. At membrane level, C3G inhibits oxidative stress-induced ROS formation and concentrates in several brain regions which are important for memory and learning such as hippocampus and cortex to protect the neurons [199]. Therefore, it is credible that C3G averts the oligomer-induced neuronal destabilization and lipid peroxidation [195]. Thus, it can serve as an alternate for the prevention of NDDs such as AD.

### 12.2. Pelargonidin

Pelargonidin (Pel) is an anthocyanin derivative flavonoid and is an agonist of ER but it possesses minimal estrogen side effects [200]. It is one of the important flavonoid which is efficiently absorbed in the gastrointestinal tract and also has an accessibility to cross the BBB [201,202]. Pel exerts a vast number of beneficial effects on human health because of its proficient absorption and minimum side effects. Being the derivative of anthocyanin, it appears to elicits a potential efficacy as anti-oxidant, anti-inflammatory [203], antihyperglycemic [204], neural protection, non-genotoxicity responses [205], and anti-thrombosis activity [206]. The underlying mechanism of its anti-inflammatory property involves the modulation of interleukins-10 (IL-10), which contributes to the protective effects in inflammatory diseases but has no effect on the IL-6, IL-1β, and IL-8 [207]. Importantly, it would be one of the most valuable substitutes to avert the age-related memory and cognitive deficits [200]. Moreover, neuroprotective property of pel in NDDs is discussed as follows.

#### 12.2.1. Pelargonidin in AD

In spite of conspicuous advances in pathophysiology and therapeutic knowledge about AD, there are only a minimum number of drugs which have been approved for symptomatic treatment due to the complex nature of the disease [208]. Anti-inflammatory agents can manage the state of disease because of their ability to modulate the underlying factors of inflammation. Likewise, Pel inhibits the inducible nitric oxide synthase (iNOS) protein and mRNA expression, NO production and NF-κB expression [209]. ERs are largely present in certain memory associated brain areas like frontal cortex, amygdala, and hippocampus [210] and they also possess neuroprotection in NDDs but the exact mechanism is not clarified yet [211]. Pel exerts its neuroprotective efficacy due to its ability to act as an agonist of ERs [212]. Blood flows in the hippocampal region may stimulate memory function and neurogenesis by its vasodilatory property. Moreover, memory concert and neuronal connectivity may also be amended by increased morphology repair and dendritic spine density in female rat models [213]. Studies have been reported that oral consumption (10 mg/kg) of Pel could converse the memory disturbance induced by Aβ_25–35_ via ERs independent pathways. Similarly, another study on rat model also depicts that it recovers the memory dysfunction in Morris water maze (MWM) test via improving the cholinergic dysfunction as well as down-regulating the glial fibrillary acidic protein (GFAP) [200]. Lastly, because of its diverse pathological mechanisms, it would be the valuable alternatives for estrogen to avert age-related memory deficit and cognitive changes in disorders like AD. However, additional studies should be done to define its precise mechanism and further explore the factors which could avert the pathogenesis of AD.

#### 12.2.2. Pelargonidin in PD

Decreased glutamate levels, oxidative stress, increased lipid peroxidation, iron deposition, and DNA damage have been reported as the major pathological factors in PD [214]. Oxidative stress impairs the dopaminergic neurons and compromises the oxidative phosphorylation of mitochondria, leading to the cell death due to insufficient availability of energy [215]. Although inordinate advances have been made in the development of medicinal therapy for PD, but none of the agent addresses the associated problem i.e., the dopaminergic neuronal damage [216]. Thus, protection of dopaminergic neuronal damage and loss is the primary need to avert the pathogenesis of PD. Pel minimizes the neuronal loss and damage via inhibiting the formation of free radicals as well as modifying the antioxidant defensive system [217]. It also decreases the formation of thiobarbituric acid reactive substances (TBARS) at the oral dose of 20 mg/kg in a semi Parkinsonism rat model whereas it is unable to prevent the free radical generation significantly at the same dose [218]. Furthermore, Pel also mitigates the development of PD because of its anti-inflammatory efficiency [209]. However, further investigation pointing the mechanistic approach of its anti-inflammatory property has to be explored yet. It may possess neuroprotective activity because of its ability to prevent the dopamine oxidation mediated by peroxynitrite. Importantly, further studies are needed regarding to its toxicity. To date, it is suggested that Pel exhibits the neuromodulatory effects because of its ability to cross the BBB and accumulates in the brain at nanomolar concentrations [219,220].

## 13. Conclusions and Future Perspectives

Neuroprotective activity of natural flavonoids encompasses multiple effects within the brain, including their efficacy to shelter against neurotoxins-induced neuronal injury, to endorse learning, memory, cognitive functions, and to suppress the neuronal inflammation. Two common processes lay the foundation of such diversified neuroprotective effects of flavonoids. Firstly, they are reported to have various positive effects on the cerebral and peripheral vascular system, leading to the alterations in cerebrovascular blood flow. These alterations ultimately induce angiogenesis, neuronal cell growth in hippocampus, and improve neuronal morphology, all of which are crucial in regulating neuro-cognitive activities and maximal neuronal functions. Secondly, they interact with neuronal signaling networks within the brain leading to the inhibition of neurotoxin-induced apoptosis and promoting the differentiation and survival of neurons.

Dietary consumption of flavonoids rich foods such as cocoa and berries grasps the efficacy to attenuate neurodegeneration and averts or reverses the age-dependent deteriorations of cognitive function. However, definite temporal nature underlying neuroprotective effects of flavonoids is unclear at present. More work is needed to be done on flavonoids as a potential therapy for several untreatable NDDs. Most particularly, at present, there are inadequate data on the aspect of a causal relationship between the consumption of flavonoids and behavioral consequences. There should be more clinical and preclinical trials. The toxic values and availability of flavonoids in the market still needs to be explored.

## Figures and Tables

**Table 1:** Classification of flavonoids and their dietary sources.

Class	Flavonoids	Dietary Sources	Diseases	References
**Isoflavones**	Genistein	Soy milk	AD, PD	[49]
	Daidzein	Soy milk	PD	[49]
**Flavones**	Luteolin	Chrysanthemum flowers, apple skins, cabbage, peppers, carrot, leaves of onion, broccoli, parsley, and celery	AD, PD	[50,51,52]
	Apigenin	Onions, parsley, grapefruit, and oranges	AD, PD	[53]
	Acacetin	Pearl millet	AD, PD	[54,55]
**Flavanone**	Hesperetin	*Citrus* species	AD	[56]
	Naringin	Citrus fruits and grapefruits	PD	[57]
**Flavanols**	(−) Epigallocatechingallate	Leaves of green tea and black tea	AD	[58]
	(−) Epicatechin	Blueberries, tea, cocoa, and grapes	PD	[59]
**Flavonols**	Quercetin	Apples, onions, tea, red wines, and berries	AD	[60,61]
	Kaempferol	Tea, broccoli, apples, beans, strawberries, and grapefruits	PD	[62,63]
**Anthocyanidins**	Cyanidin	Cranberry, blueberry, blackberry, acai berry, and raspberry	AD	[64]
	Pelargonidin	Ripe raspberry, strawberry, blueberry, cranberry, blackberry, saskatoon berry, and kidney beans.	AD, PD	[65,66]
